# Mathematical Model for Radial Expansion and Conflation of Intratumoral Infectious Centers Predicts Curative Oncolytic Virotherapy Parameters

**DOI:** 10.1371/journal.pone.0073759

**Published:** 2013-09-11

**Authors:** Kent Bailey, Amber Kirk, Shruthi Naik, Rebecca Nace, Michael B. Steele, Lukkana Suksanpaisan, Xing Li, Mark J. Federspiel, Kah-Whye Peng, David Kirk, Stephen J. Russell

**Affiliations:** 1 Department of Biomedical Statistics and Informatics, Mayo Clinic, Rochester, Minnesota, United States of America; 2 Department of Molecular Medicine, Mayo Clinic, Rochester, Minnesota, United States of America; 3 Center for Translational Science Activities, Mayo Clinic, Rochester, Minnesota, United States of America; 4 Consulpack, Inc., Minneapolis, Minnesota, United States of America; 5 Division of Hematology, Department of Medicine, Mayo Clinic, Rochester, Minnesota, United States of America; McMaster University, Canada

## Abstract

Simple, inductive mathematical models of oncolytic virotherapy are needed to guide protocol design and improve treatment outcomes. Analysis of plasmacytomas regressing after a single intravenous dose of oncolytic vesicular stomatitis virus in myeloma animal models revealed that intratumoral virus spread was spatially constrained, occurring almost exclusively through radial expansion of randomly distributed infectious centers. From these experimental observations we developed a simple model to calculate the probability of survival for any cell within a treated tumor. The model predicted that small changes to the density of initially infected cells or to the average maximum radius of infected centers would have a major impact on treatment outcome, and this was confirmed experimentally. The new model provides a useful and flexible tool for virotherapy protocol optimization.

## Introduction

Oncolytic virotherapy is an experimental cancer treatment in which viruses of evolved or engineered cancer tropisms are exploited for their ability to induce selective killing of tumor cells. Preclinical studies have demonstrated anticancer activity for a broad range of viruses in diverse tumor models, and several virotherapies have advanced to clinical trials [1], [2]. The need for mathematical modeling is readily apparent because, unlike all other established or experimental cancer therapies, oncolytic therapies amplify in the body to a variable extent after administration. Each oncolytic virus possesses unique characteristics that define how it interacts with the intratumoral environment, including extravasation, infection, replication, spread, cell killing, and immune response induction. Mathematical models which capture the resulting complexity of virus-tumor interactions have been developed to improve intuitive understanding of oncolytic therapy, predict tumor response and long-term recurrence, and guide the development of more effective oncolytic virotherapy approaches [3], [4], [5], [6], [7], [8], [9], [10], [11], [12], [13], [14], [15], [16], [17], [18], [19], [20]. Many existing models are theoretical rather than inductive, and treat infected tumors as dynamic systems with considerations for the kinetic interplay between concurrent processes of exponential virus spread, tumor growth, and the antiviral immune response [3], [4], [5], [6], [7], [8], [9], [10], [13], [15], [16], [17], [18], [20]. While some models do account for spatial constraints on virus spread, they remain complex, time dependent stochastic models [7], [12], [15], [17]. Importantly, existing models do not distinguish between wholesale tumor destruction that occurs during the oncolytic phase in which virus-infected tumor cells are killed and the residual destruction that occurs during the immunotherapeutic phase in which residual uninfected tumor cells are killed via an antitumor immune response. Therefore the aim of this work was to develop a simplified inductive mathematical model to guide virotherapy development. Our experimental evidence justifies the development of a simplified, spatially constrained model that estimates the probability of tumor cell survival solely after the oncolytic phase. The simplified model is based on the novel observation that certain virotherapies propagate very rapidly in cancerous tissue, almost exclusively through spatially-restricted spread at rates that diminish the relevance of complex population dynamics during the oncolytic phase. Therefore, this new mathematical model is designed to accurately describe observed processes of virus-induced tumor destruction prior to immunotherapeutic interactions.

## Materials and Methods

### Ethics Statement

Animals were maintained and cared for in strict accordance with the recommendations in the Guide for the Care and Use of Laboratory Animals of the National Institutes of Health. The protocol was approved by the Institutional Animal Care and Use Committee of Mayo Clinic (A23810).

### Cell Lines and Virus Manufacture

Cell lines were cultured in media supplemented with 10% fetal bovine serum (FBS), 100 U/ml penicillin and 100 mg/ml streptomycin. Vero cells were obtained from American Type Cell Culture (ATCC, Manassas, VA) and cultured in Dulbecco’s Modified Eagles Medium (DMEM). 5TGM1 murine myeloma cells were obtained from Dr. Babatunde Oyajobi (UT Health Sciences Center, San Antonio, TX USA, originally established from the parent murine 5T33 (IgG_2b_κ) myeloma [21]) and cultured in Iscove’s Modified Dulbecco’s Medium (IMDM). KAS 6/1 human myeloma cells were a kind gift from D.F. Jelinek (Mayo Clinic, Rochester, MN, originally established from myeloma patients [22]). KAS 6/1 cells were cultured in 10% RPMI-1640 supplemented with 1 ng/ml IL-6. All cell lines tested negative for mycoplasma contamination. 5TGM1 cells are syngeneic to the C57Bl6/KalwRij mouse strain and successful tumor growth confirms cell line identity. These cell lines were not otherwise authenticated.

*In vivo* studies evaluating VSV efficacy utilized VSV-mIFNβ-NIS, a recombinant VSV containing transgenes coding for murine interferon-β (IFNβ) and the human sodium iodide symporter (NIS), generated as previously described [23]. Viruses were manufactured in the Mayo Clinic Viral Vector Production Laboratory (VVPL). Viral titer was quantified by measuring 50% tissue culture infective dose (TCID_50_) on Vero cells calculated using the Spearman-Karber equation. Virus was stored at −70°C or lower until use.

### *In vivo* Studies Evaluating VSV Therapy in Murine Myeloma Models

#### Immunocompetent 5TGM1 myeloma model

5×10^6^ 5TGM1 murine myeloma cells (suspended in 100 µl sterile saline) were subcutaneously implanted in the right flank of ∼ 6 week-old female syngeneic C57Bl6/KalwRij mice (Harlan, Netherlands).

#### Immunocompromised KAS 6/1 myeloma model

1×10^7^ KAS 6/1 murine myeloma cells were subcutaneously implanted on the right flank of ∼6 week-old female CB17 ICR SCID mice. Mice received 250 cGy total body irradiation 24 hr prior to implantation.

#### Immunocompromised U266 myeloma model

p1×10^7^ U266 human myeloma cells (ATCC, Manassas, VA) were subcutaneously implanted on the right flank of ∼6 week-old female CB17 ICR SCID mice. Mice received 250 cGy total body irradiation 24 hr prior to implantation.

When tumors measured ∼0.5 cm in diameter, mice were injected with a single intravenous dose of either 100 µl sterile saline or VSV-mIFNβ-NIS at a dose of 10^5^, 10^6^, 10^7^, or 10^8^ TCID_50_. Prior to injection, virus was diluted in sterile saline to required dose for 0.1 ml total injection volume per mouse. Tumor response was monitored by serial caliper measurements in two dimensions and tumor volume was calculated using the formula V = 0.5*a^2^b* (where *a*≤*b*). Mice were euthanized if tumors reached greater than 10% of mouse body weight, if tumors were severely ulcerated, if weight loss was greater than 20%, or if mice were unable to access food and water or were in obvious distress.

Tumor volume was used to determine therapeutic response. Response was defined as a reduction in tumor size at day 12 post treatment relative to tumor size at day 0, both for individual mice and for average tumor size of treatment groups. Partial response is defined as ≥50% reduction in tumor volume compared to day 0.

### High-resolution Immunofluorescence Tumor Analysis

5TGM1 tumors were implanted in syngeneic mice as previously described. Mice were treated with a single intravenous dose of sterile saline, 2×10^8^ TCID_50_ VSV-mIFNβ-NIS, or 1×10^7^ TCID_50_ spread-deficient VSV(ΔG). Tumors from treated mice were harvested at various time points ranging from 6 hr to 72 hr post injection and frozen in optimal cutting medium (OCT) for sectioning. Tumor sections were subject to immunofluorescence staining for (i) VSV antigens (red for 5TGM1 and KAS 6/1 models, green for U266 model) using a primary rabbit polyclonal antibody generated by the Mayo Clinic VVPL followed by an Alexa-labeled anti-rabbit secondary antibody (Invitrogen, Molecular Probes) (ii) cell death using TUNEL (Terminal deoxynucleotidyl transferase dUTP nick end labeling) staining (Promega) and (iii) cellular nuclei using Hoescht 33342 (Invitrogen).

Intratumoral VSV extravasation was visualized by harvesting tumors 24 hr post intravenous administration of spread-deficient VSV(ΔG), which undergoes a single cycle of infection and cannot undergo subsequent rounds of infection, allowing visualization of cells infected upon viral extravasation within a 24 hr period. Expansion of intratumoral foci of infection was quantified in tumor sections harvested at 6 hr intervals following intravenous spread-competent VSV administration, and sections were subject to staining for VSV antigens. Intratumoral foci were quantified by measuring 7–8 foci from multiple sections from two tumors harvested at each time point. Each focus diameter was divided by average tumor cell diameter (measure as an average diameter of 50 individual tumor cells) to obtain focus diameters in number of cells. The volume of each approximately spherical focus was estimated using the formula *V = (4/3)π(d/2)^3^* expressed as number of cells and plotted versus time. The rim of viable infected cells was similarly quantified by measuring approximate rim width at the advancing edge of infection from three tumors harvested 48 hr post VSV administration (difference between advancing edge of anti-VSV staining and advancing edge of TUNEL staining). This width was divided by average cell diameter to approximate the number of cells that are progressively infected before cells succumbed to cell death.

### Statistical and Analytical Methods

Tumor growth curves were generated to compare tumor growth and response in C57Bl6/KalwRij mice bearing 5TGM1 tumors and CB17 ICR SCID mice bearing KAS 6/1 tumors after single IV administration of VSV-mIFNβ-NIS at doses of 10^5^, 10^6^, 10^7^, or 10^8^ TCID_50_ compared to saline treated mice using Prism® (GraphPad, Software Inc., La Jolla, CA).

Expansion of intratumoral foci and tumor growth was fitted to the exponential growth equation [Y = Y0*e^(k*X)^] using Prism® to obtain an approximate doubling time and R^2^ value.

The mathematical model derived here was analyzed for relationships between *k*, *R*, and *r* using mathematical software packages. Mathcad® 7 Professional (PTC, Needham, MA, USA) was used to analyze integrals for 100 values of *K* and 100 values of *r/R* to generate a matrix of 10,000 data points describing relationship between the above variables. The matrix was then analyzed using Excel 2010® (Microsoft Corporation, Redmond, WA, USA). Further data manipulation, analysis and visualization, including heatmaps and 3-D perspective plots, were performed using custom R scripts with R programming language.

## Results

**Figure 1:**
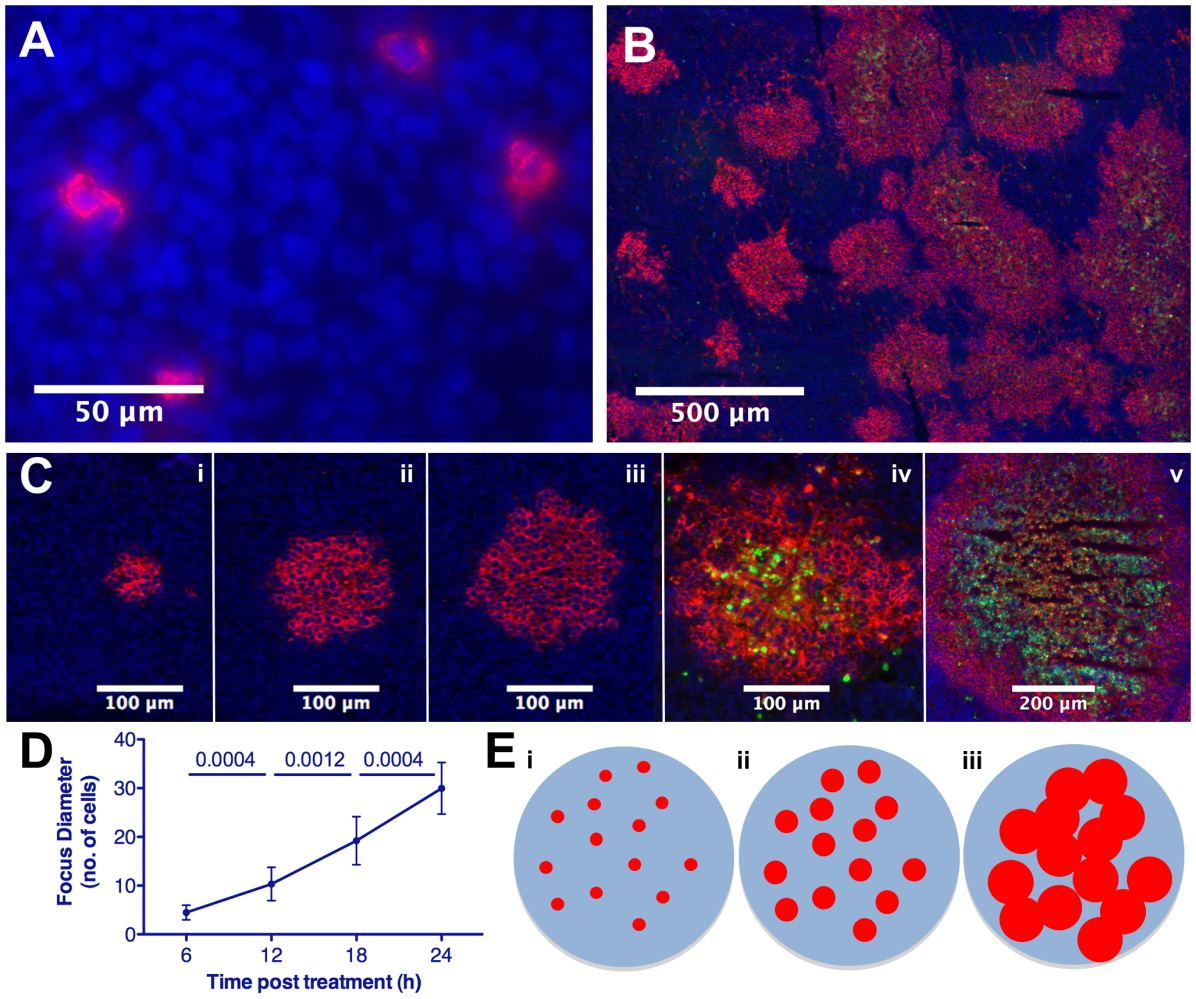
Extravasation and spatially constrained spread of systemic oncolytic therapy. Immunofluorescence analysis and quantification of 5TGM1 tumors harvested following IV VSV administration, sectioned and stained to detect VSV (red), dying cells (TUNEL, green) and tumor cell nuclei (Hoescht, blue). (A) Seeds of infection established following virus extravasation 24 hr post-VSV(ΔG). (B) Expansion and conflation of intratumoral foci and destruction of tumor cells 48 hr post-VSV. (C) Radial expansion of infection and subsequent cell death of intratumoral focus in tumor harvested at 6, 12, 18, 24, and 48 hr post-VSV(i-v). (D) Quantification of mean infectious focus diameter (n = 7–9/interval) in tumors harvested at 6 hr intervals post-VSV. (E) Schematic representation of proposed model of systemic oncolytic therapy showing (i) extravasation and infection of tumor cells seeding randomly distributed infectious centers, (ii) spatially constrained expansion, and (iii) conflation of foci resulting in viral destruction of tumor cells, though voids of uninfected, surviving cells remain.

We previously showed that plasmacytomas regress after a single intravenous (IV) administration of oncolytic vesicular stomatitis virus (VSV) [23]. To investigate intratumoral viral extravasation in the absence of spread, a spread-defective VSV missing a critical gene (VSVΔG) was administered intravenously and immunostaining of tumor sections 24 hr later revealed single infected tumor cells dispersed throughout the tumor parenchyma (Fig. 1A). Immunohistochemical analysis of tumors explanted 6, 12, 18, 24 and 48 hr post IV administration of spread-competent oncolytic VSV showed a striking pattern of radially expanding, roughly spherical, infectious centers (Fig. 1B, C). Virus transmission is spatially constrained, expanding outward from initially infected cells to uninfected tumor cells. Cell death follows infection, and initially infected cells die first followed by more recently infected cells near the perimeter (Fig. 1C). Consequently, curative plasmacytoma regression due to oncolytic VSV therapy is the result of rapidly expanding infectious centers in which infected cells die, and foci in close proximity conflate causing massive tumor destruction and complete regression at high doses during the oncolytic phase (Fig. 1B, C, E).

**Figure 2:**
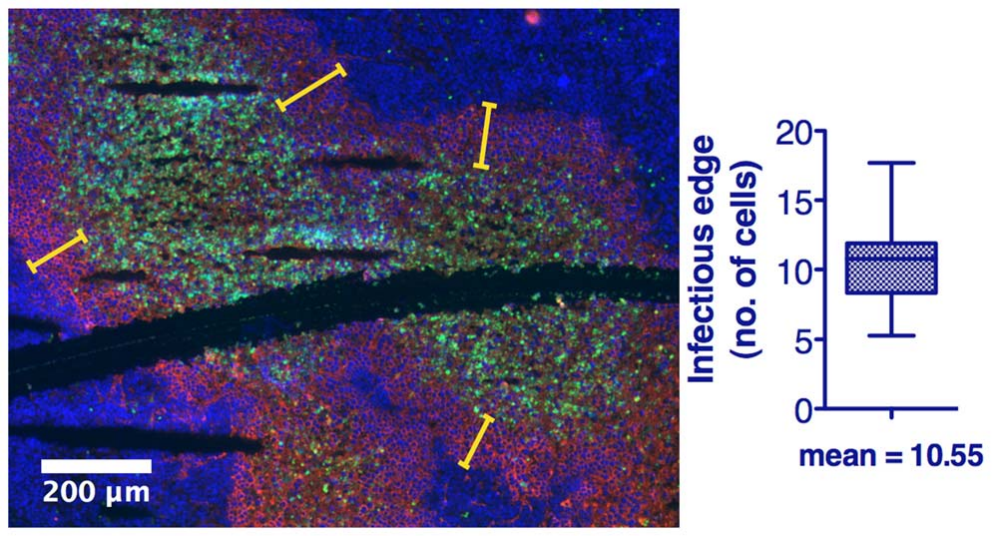
Quantification of viable infected rim at the leading edge of infection to determine cell death rate. Immunofluorescence analysis of 5TGM1 tumors harvested 48-VSV administration, sectioned and stained to detect VSV (red), dying cells (TUNEL, green) and tumor cell nuclei (Hoescht, blue). Quantification of the mean viable rim width(n = 36 measurements), expressed in terms of cell diameters, at the leading edge of infection allows for the time from cellular infection by the VSV to cell death to be determined. Yellow bars indicate example locations of rim width determination.

**Figure 3:**
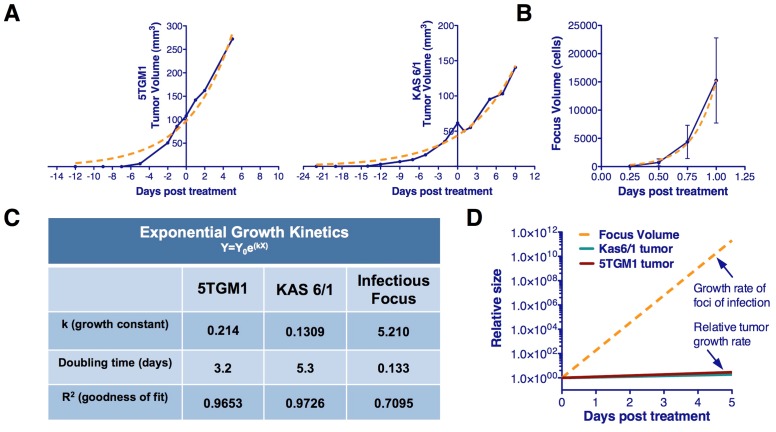
Tumor growth rates are negligible in comparison to growth of infectious foci. Kinetics of tumor growth. (A) Subcutaneous 5TGM1 or KAS 6/1 myeloma tumors were measured by serial caliper measurements following implantation in immunocompetent C57Bl6/KaLwRij or immunocompromised SCID mice respectively. Volume was calculated using the formula (l*h^2^/2). Tumor growth was fitted using the exponential growth equation Y = Y_0_e^(kX)^, where tumor doubling time was approximately 3.2 and 5.3 days respectively. (B) Kinetics of intratumoral infectious foci expansion based on average infectious focus volume determined by the equation V = 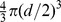 where *d* is the focus diameter (Fig. 1D). Focus expansion was fitted using the exponential growth equation Y = Y_0_e^(kX)^, where focus doubling time was approximately 3.2 hours. (C) Exponential growth rate, doubling time and goodness of fit for approximated growth curves of tumor models and infectious focus expansion. (D) Comparison of infectious foci expansion rate and subcutaneous 5TGM1 of KAS 6/1 myeloma tumor growth rates relative to size of tumor at time of virotherapy administration.

Quantification of immunofluorescence data showing viral spread revealed an approximate radial expansion rate of ∼0.7 cells/hr during the first 48 hrs, by which time foci reach maximum size and coalesce with nearby foci, often becoming indistinguishable from nearby foci (Fig. 1B, 1D). A rim of viable infected cells at the advancing edge of infection averaged 10.55 cells in width (Fig. 2). Thus, the time from cellular infection to death is approximately 15 hours. This rate is much greater than that of implanted 5TGM1 or MPC11 tumor growth which can therefore be assumed negligible during the oncolytic phase (Fig. 3). The contributions of adaptive antiviral and antitumor immune responses, the latter dependent on cross-priming of reactive T-cells in the context of the antiviral response, are also assumed negligible during the initial oncolytic phase of the therapy as they occur after the initial phase of tumor destruction caused by the established infectious foci. Anti-VSV neutralizing antibodies are not detected in serum until day-5 post VSV administration in C57Bl6/KaLwRij mice bearing subcutaneous syngeneic 5TGM1 myeloma tumors, confirming that the rapid spread of viral infection that creates the infectious foci modeled here is completed before antiviral antibodies become detectable in the bloodstream [24].

The innate antiviral response, including cellular interferon responses and tumor resident inflammatory cells, is recognized as an important factor limiting intratumoral virus spread [25]. We also recognize that infiltrating neutrophils and tumor vascular shutdown can limit viral spread, although those are not seen in this model [23]. The model, looking at a distinct time after foci spread, accounts for all innate immune response components, in addition to all other virus-tumor interactions that contribute to kinetics of replication and spread, in a single variable describing final focus size so that innate immune responses do not need to be considered separately. Based on these observations, the following assumptions allowed for a simplistic, easy to use mathematical model of oncolytic virotherapy to be developed.

### Model Assumptions

The following assumptions were made for the purpose of modeling the probability of tumor cell survival at a fixed time point after systemic administration of an oncolytic virus. We recognize that these are assumptions and will address modifications to these assumptions in the discussion.

#### Virus delivery

Intravenously administered virus extravasates randomly (Poisson distribution) throughout the entire tumor so that only the density of focus initiating cells must be accounted for. Infection of normal cells is neglected due to natural tumor tropism of oncolytic viruses.

#### Virus spread

Virus spreads only to cells in direct vicinity of an infected cell, such that infection radially expands at a predictable rate (time of transmission ∼0.7cells/hr, Fig. 1D) and decelerates and stops at a predictable time due in part to innate antiviral immune response and interactions with tumor microenvironment. At this time, each infectious center is static and has reached an equal maximum size, and there is no other mode of virus spread (e.g. secondary viremia).

#### Tumor cell killing

All infected cells die. All uninfected cells survive the oncolytic phase.

#### Tumor growth

Tumors are spherical and growth is exponential with an empirically determined doubling time which is sufficiently slow compared to virus spread so that it can be assumed that the rate of tumor cell division is negligible with respect to the rate of virus spread and is therefore neglected (Fig. 3).

#### Immune response

Innate immunity in part determines the maximum focus size and is therefore not considered independently. Changes in adaptive immune response do not occur during the period of virus spread [24], and is therefore neglected during the modeled oncolytic phase that occurs prior to adaptive response.

### Mathematical Modeling

The mathematical model assumes a spherical tumor of radius *R* is subject to *K* infection foci (determined by initially infected cells dependent on viral dose and extravasation) distributed independently and randomly (Poisson distribution) inside the tumor. If each focus grows to an equal maximum radius, *r,* the probability of survival at a given intratumoral location–the probability that all *K* foci of radius *r* “miss” a given point of consideration “*x_o_*”– can be determined. The average survival probability for all points *x_o_* within the tumor gives the predicted proportion of the tumor surviving and is defined by the mathematical model derived here.

**Figure 4:**
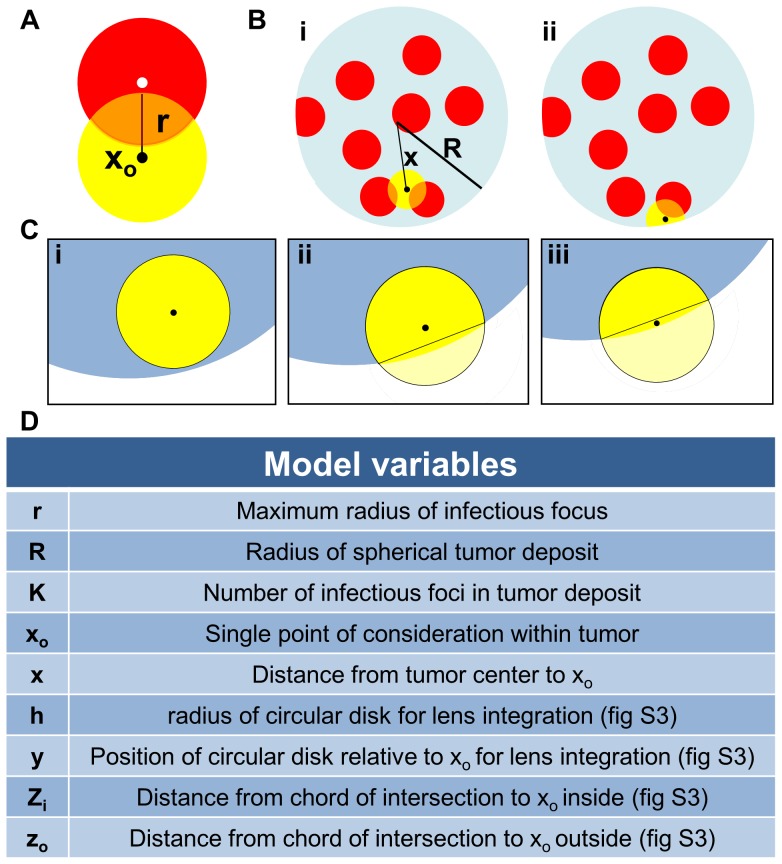
Diagram of mathematical model variables. (A)Volume of vulnerability (yellow) is defined as the volume surrounding *x_o_* that must be void of focus initiating cells (white) to remain uninfected after oncolytic viral spread (red). (B) Diagram of a spherical tumor depicting foci of infection (red) and (i) a spherical volume of vulnerability (yellow) for the tumor interior and (ii) a lens of vulnerability (yellow) near the tumor periphery. (C) Three unique volumes of vulnerability defined by the value of *x*; (i) a complete intratumoral sphere when 0≤*x*≤*R-r*, (ii) a lens formed when the center of the sphere of radius *r* lies inside of the chord of intersection, *R-r*<*x*≤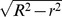, and (iii) a lens formed when the center of the sphere of radius *r* lies outside of the chord of intersection, 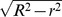
<*x* ≤*R*. (**D**) A table of model variables.

This very high resolution *in vivo* experimental data sheds new light on the mechanism of intratumoral virus spread that occurs almost exclusively through radial expansion of randomly distributed infectious centers generating spherical foci of virally infected cells allows consideration of tumor survival as a static geometric problem. We begin by calculating the probability that a single randomly distributed infection sphere fails to include *x_o_* by considering that *x_o_* is surrounded by a volume of vulnerability. A volume of vulnerability can be determined by noting that any infection that initiates within a distance *r* from *x_o_* will create an infectious focus that includes the point *x_o_* after viral spread, thereby making *x_o_* vulnerable to infection (Fig. 4A). Hence, the volume of vulnerability defines the volume required to be void of infection-initiating centers. Rather than modeling infectious spheres, we have generated this negative space model that allows for simplified probabilistic modeling.

The volume of vulnerability for *x_o_* is defined as a function of the distance of *x_o_* from the center of the tumor, *x*, and is a complete intratumoral sphere of radius *r* when 0≤*x*≤ *R-r* (Fig. 4B). Near the tumor periphery, (*R-r)*<*x*≤*R*, the spherical volume of vulnerability intersects the tumor sphere, creating a lens of vulnerability contained within the tumor rather than a sphere (Fig. 4B). This loss of volume of vulnerability at the tumor periphery has been termed the edge-effect.

Lens volume can be determined by integrating the volume of circular disks over the span of the lens, as determined in two distinct cases: *x_o_* can be positioned inside or outside of the chord of intersection created by the intersection of the spheres of radius *R* and *r* relative to the tumor center, creating the interior lens when *R-r*<*x*≤
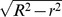
and the outer lens when 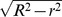
<*x*≤*R* (Fig. 4C, Table 1). The interior lens volume is determined by first defining the distance of *x_o_* from the chord of intersection, *z_i_*, in terms of the tumor radius, *R*, focus radius, *r*, and distance from the center of the tumor, *x.* We then integrate the volume of circular disks of height *h*, with thickness of *dy* over the entire lens when *–r<y<z_i_* and for *z_i_<y<R* where *y* is the position along the same axis as distances *x* and *z* relative to the position of *x_o_* (File S1, Fig. S1, Table 1)_._ The similar formulation is done for the outer lens volume, using the distance from the chord of intersection *z_o_* integrated over the entire lens when *–(r+z_o_)<y<-z_o_* and for –*z_o_<y<R*. (Fig. S1, Table 1)_._

**Table 1:** Components of mathematical model derivation.

	Interior case0≤*x*≤ *R-r*	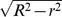 Inner lens*R-r*<*x*≤	Outer lens 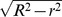 <*x*≤*R*
Volume of vulnerability	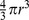	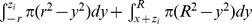	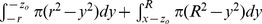
Proportion of tumor vulnerable	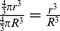	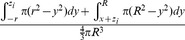	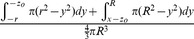
Proportion not vulnerable	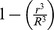	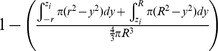	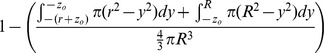
Probability of escaping *K* foci	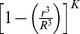	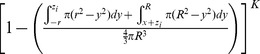	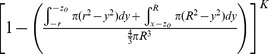
Probability of consideration and escaping *K* foci	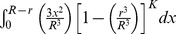	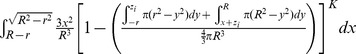	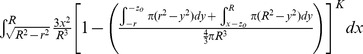

The volume of vulnerability can be expressed as a proportion of total tumor volume (Table 1). The proportion of tumor outside this volume of vulnerability remains open to focus initiating infection and is equivalent to the probability of *x_o_* escaping a single infectious focus (Table 1). Following rules of multiplicity, this can be expanded to include escape of *K* infectious foci (Table 1). Given that the probabilities of survival are functions of distance from the center of the tumor, we must calculate and analyze this probability for all points *x_o_* as a function of all distances *x*. The probability of having a point of consideration *x_o_* at a distance *x* in any direction from the tumor center varies with respect to *x* such that the probability of considering a point positioned at a small value of *x* away from the center is less than considering a point positioned at a greater value of *x* away from the center. We can define this probability using an integral in which we consider the volume of a spherical shell with a thickness of *dx* with respect to the total tumor volume.
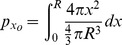



We then integrate the probability of being positioned at a distance *x* multiplied by the probability of survival for points at that distance *x*, distinctly defined for 3 regions: interior, inner lens, and outer lens. This yields 3 integrals that effectively describe the probability of any point being located within each distinct region and escaping *K* infectious foci (Table 1). The summation of these integrals yields the average survival probability for any point within the entire tumor. 
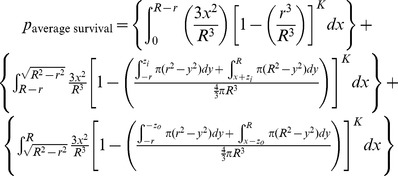


This probability will define the average probability that any single point within the tumor will survive after oncolytic phase of systemically delivered VSV.

In order to simplify this model, we recognize that the outer lens volumes are vanishingly small, especially in instances when *R*>>*r*. This allows us to simplify the volume of intersection for foci at the tumor periphery. When we assume the curvature of the sphere radius *R* is negligible within the overlapping region with sphere of radius *r*, we can approximate the volume outside of this intersection to be a spherical cap (Fig. S2). This allows the volume of vulnerability to be defined without integration (Table S1, [26]). The corresponding manipulations can be performed as above to derive the probability of avoidance of *K* infection spheres (Table S1).

The probability of escaping *K* infectious foci for *x_o_* at distance *x* from the center of the tumor is then integrated over all values of *x* and weighted by the proportion of the tumor sphere located at distance *x* from the tumor center, to give the approximate average probability of any point within the tumor surviving after the oncolytic phase of systemically delivered VSV.
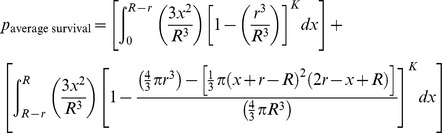



If it is assumed that the difference in volume between the lens of vulnerability and the corresponding sphere of vulnerability in the border case is minimal, the model can be further simplified to neglect the edge effect altogether. Then we are only concerned with the number and size of foci. This allows the model to simply describe relationships between viral distribution, in terms of *K*, and infectious center size, *r*, on the resulting survival probability of the tumor.
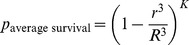



Both approximations can be used without substantial loss in the model’s predictive power (Fig. S3). This final model approximation provides a simple alternative way of conceptualizing oncolytic virotherapy that will be of practical use as it distills optimization of viral parameters to two key issues; increasing viral distribution and increasing viral spread.

**Figure 5:**
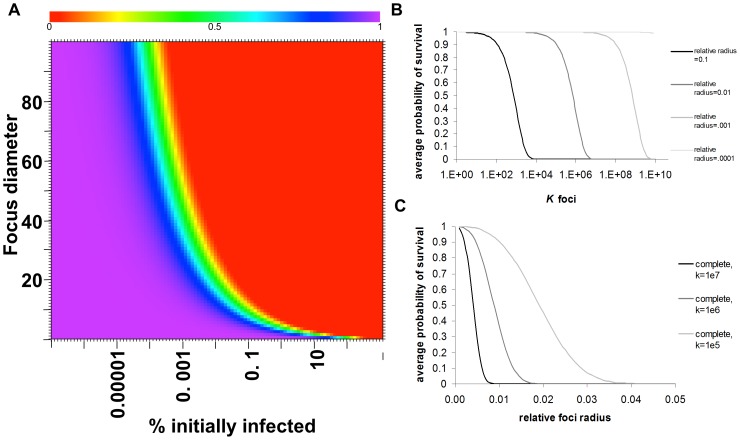
Math modeling of oncolytic tumor destruction and experimental validation. (**A**) Heatmap depicting average probability of tumor survival with respect to modeling parameters *r/R* and *K* determined using the spherical-cap approximation. Values of *r/R* and *K* have been converted to standard units of focus diameter (cell diameters) and percent of tumor cells infected at time zero of foci expansion respectively. Red = 0.00 probability of survival. Purple = 1.00 probability of survival. Sharp gradient from purple to red reveals drastic drop in survival upon small change in viral parameters. This gradient defines threshold of therapeutic efficacy. (B) Dose-response relationship: When the relative size of foci radius is kept constant, small changes in foci density, *K*, cause drastic changes in survivability beyond threshold dose. (C)When the density of foci is kept constant, small changes in the relative radius of infection foci cause changes in survivability. An increase in relative foci size achieves greater survivability at greater doses.

The complete mathematical model or the model approximations can be evaluated for relationships between *K, R*, and *r* to determine parameters necessary for tumor cure following the oncolytic phase of VSV. The model can be graphed two- and three-dimensionally to yield a heatmap (Fig. 5) and surface plot of survival probability respectively (Fig. S4). These visualizations provide insight into therapeutic optimization by defining the relationship between foci density, viral spread, and changes in tumor survival. Most significantly, the model reveals a threshold of therapeutic efficacy that is dependent on both viral dose and distribution and viral spread. Small changes in density or size of infectious centers result in drastic changes in survival probability. Prior to the threshold, tumor survival is very high. Beyond this threshold, tumor survivability dramatically and rapidly decreases. This dramatic change in survivability occurs within 2 logs of foci density; for any focus diameter, tumor survivability drops from 100% to 0% over an approximate 100 fold increase in density above the threshold density. The dramatic change in survivability can also be achieved by increases in viral spread. For initially very small, high density foci, the drop in survivability due to increased viral spread is very rapid. As the threshold shifts with increasing focus size or decreasing density, the change in viral spread necessary to achieve a substantial drop in survivability increases, and the influence of viral spread plateaus. Therefore limits of therapeutic efficacy are reached for both increases in *r* or *K,* beyond which minimal increases in efficacy are gained.

### Model Validation

**Figure 6:**
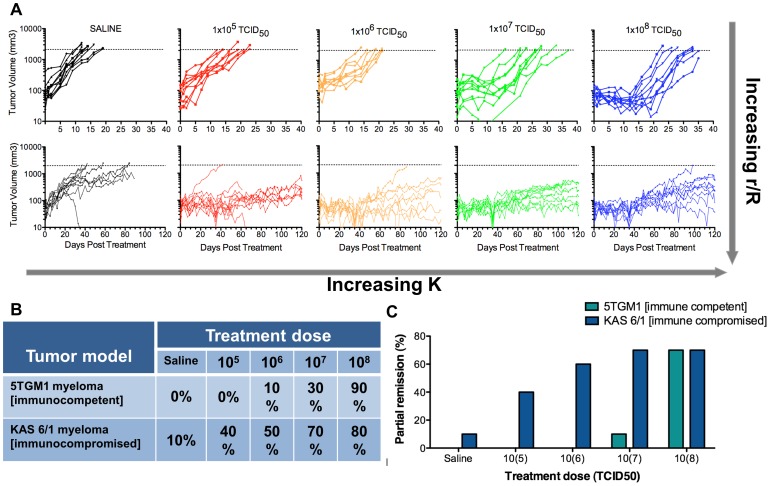
Dose escalation in immunocompetent and immunocompromised myeloma tumor models validates model predictions. (**A**) Tumor volume monitored by serial caliper measurements in C57Bl6/KaLwRij mice bearing 5TGM1 tumors after single IV administration of sterile saline or VSV-mIFNβ-NIS at doses of 10^5^, 10^6^, 10^7^, or 10^8^ TCID_50_ (top) and in CB17 ICR SCID mice bearing KAS 6/1 tumors after single IV administration of sterile saline or VSV-mIFNβ-NIS at doses of 10^5^, 10^6^, 10^7^, or 10^8^ TCID_50_ (bottom) is plotted against time. Dotted line represents tumor volume sacrifice criteria. Dose escalation is used to model increasing *K*. Immunocompromised mice allow for extended periods of infectious foci expansion, increasing *r/R*. The model is validated by (B) dose-dependent tumor response, defined as proportion of mice with tumor regression at day 12 relative to baseline, and (C) partial remission, defined as tumor regression ≥50% compared to baseline after a single IV injection of VSV-IFNß-NIS in immunocompetent C57Bl6/KaLwRij or immunocompromised SCID mice bearing subcutaneous plasmacytomas.

The model predicts increased density will cause increased tumor destruction, measured by tumor regression. Since virus dose drives the density of infected cells, the model predicts a dose-response relationship (Fig. 5B). To validate this relationship, immunocompetent mice bearing 5TGM1 myeloma tumors were administered log-fold increasing viral doses. As predicted, increasing viral doses (increasing *K*) had progressively greater impact on tumor regression. Minimal tumor regression occurred at doses up to 10^6^ TCID_50_ while doses above this level showed increasing tumor response, and profound regression occurred at a dose of 10^8^ TCID_50_ (Fig. 6). Slight increases of the dose above 10^8^ TCID_50_ resulted in a high frequency of complete cure [23]. To test the prediction that increasing infectious center size could also have a major impact on the probability of tumor regression (Fig. 5C), escalating doses of virus were administered to immunocompromised SCID mice bearing subcutaneous plasmacytomas. In these immunodeficient mice, expansion of infectious centers is not constrained by the immune system, substantially increasing *r*. In line with the model predictions, compared to the responses seen in the immunocompetent plasmacytoma-bearing mice, similar responses to treatment in the SCID mice were achieved at 100-fold lower virus doses (Fig. 6). Furthermore, efficacy is dependent on the oncolytic therapy and tumor type, both of which are reflected by the experimentally determined variable *r*. Different tumor systems with comparable focus density but vastly different maximum focus diameters showed regression in tumors with large foci and no regression in tumors with small foci (unpublished data), further demonstrating the influence of foci diameter (Fig. S5). This also demonstrates the flexible nature of this model to portray different tumor-virus systems through the manipulation of simple variables. These studies validate the predictive power of the model, particularly with respect to the effects of dose-escalation and extended focus expansion on oncolytic efficacy.

## Discussion

The simplistic mathematical model presented here provides a new approach to optimizing oncolytic virotherapies that is both practical and pertinent. The model treats oncolytic virotherapy as a two phase system in which the efficacy of the first phase is dependent on viral infection, spread, and infected cell oncolysis. It is novel as it presents a refined and simplified method of predicting tumor response to systemic oncolytic viral therapy that was derived from experimental observations. These experimental observations allowed this model to be set apart from previous models for fundamental reasons- it depicts systemic therapy, distinguishes efficacy gained in the oncolytic phase, justifies simplicity, and highlights the significance of the virotherapy dose response. This model is able to generate predictions about tumor eradication that align with existing literature but is able to do so based on novel insights that simplify the approach to therapeutic optimization by reducing the parameters of consideration to two fundamental parameters; viral distribution and viral spread. Furthermore, the model is malleable to fit new experimental observations and different tumor systems.

Previous mathematical models, although informative, are theoretically derived and then fit to experimentally observed parameters [3], [4], [5], [6], [7], [8], [9], [10], [13], [15], [16], [17], [18], [20]. Conversely, this model was developed from *in vivo* experimental observations. This approach allowed for the differentiation of therapeutic phases due to data that revealed the rapid speed of viral spread relative to the rate of tumor growth or adaptive immune response results in the formation of finite foci of infection at the end of the oncolytic phase of therapy. This justifies the exclusion of complex population dynamics considered by theoretical approaches, as they occur in the later immunotherapeutic phase [3], [5], [10], [11], [12], [13], [15], [16], [17], [18], [19], [20]. The current model is thereby simplified to predict therapeutic parameters to achieve cure prior to the involvement of complex population dynamics. This simplification is applicable to all scenarios in which the rate of virus spread is much greater than the rate of tumor cell growth or adaptive immune response and when virus spread achieves stable infection foci.

This simplicity extends to the resulting predictions. Previous models estimate probability of time-dependent tumor cell survival and estimate resulting tumor size with respect to time post treatment. These outcomes rely on the time-dependent dynamics that emerge from the interactions between the virus and tumor cell populations [3], [4], [5], [7]. Neglecting the impact of time by differentiating therapeutic phases reduces the complexity of the model prediction.

Our model agrees with previous models that show the efficacy of virotherapy to be determined by entry efficiency and resulting location of infection, replicative capacity, and ability to spread, all of which determine the extent of damage incurred during the oncolytic phase [9], [12], [13], [14], [15], [17], [18], [19], [20]. Previous models predict that the location of infectious centers occurring from intratumoral injections and subsequent virus spread or extravasation within the tumor effect tumor destruction [12], [15], [19], [20]. This supports our consideration of edge effects on virotherapy efficacy. However, these models do not accurately portray patterns of delivery achieved by systemic therapy. The importance of viral spread kinetics on therapeutic outcomes have also been supported previously, however it was not recognized that viral spread stops, producing stable infectious foci [9], [10], [13], [18]. Our model is different in its ability to take into account focus-initiating infection and subsequent spatially-restricted spread at all locations of the tumor while maintaining the influence of infectious center location. Lastly, the dose- response and therapeutic threshold predicted by our model is consistent with other work [14], [18]. The novelty of our insight is in the drastic nature of the predicted dose response above therapeutic thresholds. Small changes in virotherapy protocol can lead to extreme changes in oncolytic phase efficacy.

Our model also illustrates the important interplay between changes in dose and viral spread that will lead to curative therapeutic parameters. This reconciles discrepancies between previous models. While previous models disagree whether increases in viral infection, spread, kill rates, and immunomodulation impede or improve therapeutic efficacy [7], [8], [9], [10], [11], [14], [18], it appears an adequate balance between these parameters is most efficacious. With this model, many of these interactions are defined in a single experimentally observed term. The maximum infectious radius *r* inclusively accounts for the influence of viral spread and cell death rates, burst size, and innate immunity. Additionally, a dose response relationship exists between viral dose and focus-initiating centers (*K*) that directly impacts therapeutic efficacy. Therefore, an optimal balance of *K* and *r* will lead to maximum oncolysis, shown experimentally by modifying virus dose and immune response respectively. Previous assumptions that because the virus replicates, dose does not matter is squelched by this model. The model underscores the critical importance of dose escalation and developing drugs to combat innate immune responses to allow the intratumoral infectious foci to grow to a larger average size. The results of these *in vivo* experiments are in agreement with the predictions and assumptions of the model, providing important experimental validation.

These predictions are in alignment with existing model predictions, and to put our work in the context of existing literature, our results are compared with the predictions of previous mathematical models, specifically three recent models that also focus on spatially constrained viral spread, Wodarz et al [17], Mok et al [12], and Wein et al [15]. These existing models, although complex in nature, support our model assumptions and predictions.

Wodarz and colleagues [17]used a recombinant adenovirus in an *in vitro* setting to observe spatial patterns of virus spread and then used an agent-based stochastic computational model to predict survival. In their model time-dependent and probabilistic stochastic events are maintained while contributions of complex factors such as immune system, tumor vasculature, and physical barriers are neglected at a theoretical level. Our model also neglects such contribution on the basis of experimental observations of viral kinetics that justify such neglect. Like our *in vivo* observations, the authors saw in the two-dimensional setting that virus was transmitted to uninfected cells in the immediate vicinity of infected cells, forming three distinct patterns of spread. In our three-dimensional *in vivo* models we also saw this spatial constraint of virus spread to the immediate vicinity of originally infected cells such that foci of infection are formed. Our observations most closely resembles the “hollow ring” pattern observed by Wodarz et al, however our observations allow for simplified modeling as only one pattern of viral spread was observed. Our observations not only support the theoretical neglections made by the authors, but also allow added simplicity relative to Wodarz et al. as only one pattern of spatial restricted viral spread was observed. Their prediction that the hollow ring structure is formed when the virus outruns the antiviral factors is in support of our experimental observations and subsequent modeling assumptions that virus spread is sufficiently fast during the oncolytic phase that other complex population dynamics are moot during this phase.

A model by Mok et al [12] also considers complex population dynamics in a stochastic model of intratumoral injected HSV virotherapy. This model considers complex parameters of therapy including tumor permeability and viral diffusion and binding kinetics, all of which are taken into account by *r* in our model. The overall conclusion is that improving penetration and diffusivity of HSV in tumors will increase efficacy. Our model, based on the assumption of random viral distribution within the tumor from systemic delivery, predicts that oncolytic virotherapy will be able to achieve cure as long as *r* and *K* are sufficient, which we also acknowledge will benefit from increased distribution of virus in the tumor. Another prediction of our model relates to increases in *r* through immune suppression and is consistent with the model by Wein et al [15] who state that in addition to diffuse intratumoral infection, tumor eradication by oncolytic adenovirus will probably require potent suppression of innate immune clearance mechanisms. This model uses intratumoral injection of a replication competent virus that is able to spread infection to nearby cells, presenting patterns of viral spread that is also spatially contained. This complex model comprises a system of partial differential equations that follows five distinct entities within the tumor while we are able to simplify the system to two discrete entities at the end of oncolysis-uninfected tumor cells and infected tumor cells. The authors predict threshold conditions for tumor clearance in the absence of immune mediated clearance, consistent with the therapeutic threshold demonstrated by our model, although we provide direct evidence to substantiate the absence of immune contributions. Their model implies that tumor is eradicated if virus infection can outpace the tumor proliferation, consistent with our model assumptions and observations. The authors conclude that tumor eradication will require a diffuse pattern of infection within the tumor mass, that the rate of viral spread is critical, and immune-mediated viral clearance must be suppressed. The conclusions from this complex model of intratumoral injection of oncolytic virus are consistent with our simple model’s predictions regarding the importance of viral distribution (*K*) and viral spread (*r*) and support the use of systemically delivered virus. Our model aligns nicely with previous models both in the support our experimental observations lend to previous model assumptions regarding contributions of immune components and in the consistency of the resulting predictions regarding the importance of disperse viral distribution and enhanced viral spread.

Lastly, our model provides a starting point for future experimentation and is flexible to be adjusted for different experimental systems. The model is based on a series of assumptions that are approximations regarding viral and tumor parameters that can be refined through future experimentation in order to more accurately reflect the biology of the system while maintaining the model’s simplistic nature. The assumption that *K* infected cells are distributed randomly may be an oversimplification, as virus extravasation from tumor blood vessels is impacted by regional variations in intratumoral vascular permeability, blood flow, and interstitial fluid pressure [27], [28], [29]. Future refinements could account for variability in extravasation, effectively helping to refine the volume of vulnerability or the distribution of this volume in cooperation with the proposed method. The uniform maximum focus size is an approximation of mean focus radius within a normally distributed sample. It is dependent on the specific oncolytic therapy and tumor type, both of which are reflected by the experimentally determined variable *r*. The assumption that tumor growth rate is negligible with respect to the rate of virus spread during the oncolytic phase was based on experimental evidence (Fig. 3). We recognize that regional variations in tumor cell growth rates could impact therapeutic outcomes estimated by this model. For example, it is unknown whether tumor cell growth takes place predominantly at tumor center or periphery, which is important when considering modeled edge effects [30]. For scenarios based on slower replicating viruses, inclusion of tumor growth during the oncolytic phase may be a necessary refinement. Adaptive antiviral and antitumor immune responses after initial oncolytic phase that can hinder or improve oncolytic therapy outcomes add further complexity [25], [31], [32]. They can be addressed in future studies where the current model can be modified to describe adaptive immunological responses and subsequent tumor growth responses beyond the oncolytic phase. In this way, the model maintains simplicity while remaining flexible to adjustments reflecting observed viral parameters.

To summarize, this novel spatial mathematical model predicts the probability of tumor cell survival after a single IV administration of oncolytic VSV. The model is based on immunofluorescence analysis of intratumoral virus propagation and is experimentally validated by detailed dose-response relationships in myeloma models. In contrast to previous models, the new model is explicitly spatial, neglecting all dependencies on time or population dynamics due to evidence showing virus spread occurs at rates much greater than tumor cell growth or immune modulation. While other models predict future survivability or recurrence rate, the new model aims only to predict probability of any tumor cell surviving after the oncolytic phase of a single dose administration and will therefore be useful in optimization of single-shot curative therapies [23].

## Supporting Information

Figure S1 **Modeling parameters for determination of lens of vulnerability volume.** The volume of the lens created by the overlap of spheres of radii *R* and *r* is determined for two unique cases, (A) when *x_o_* lies inside the chord of intersection and (B) when *x_o_* lies outside the chord of intersection but within the tumor. Lens volume is determined by the sum of two spherical cap volumes using integration of circular disks of radius *h* and height *dy* for all values of *y* for each cap; (i) one cap generated by the curvature of sphere radius *r* and the chord of intersection, and (ii) one cap generated by the curvature of sphere radius *R* and the chord of intersection.
(TIF)

Figure S2 **Mathematical model diagram of spherical cap approximation**. Diagram of a spherical tumor depicting volumes of vulnerability for points within the interior and border case. Inset depicts the spherical cap approximation for calculating edge-effect. The model is presented as the average survival of any point *x_o_* in the spherical tumor radius *R* after expansion of *K* foci to maximum radius *r*. A simplified model neglecting edge-effect completely can be written in terms of foci per unit volume, *d*, and maximum radius *r*.
(TIF)

Figure S3 **The influence of edge effect on model prediction of tumor survivability becomes increasingly negligible as viral parameters r/R and K increase.** Comparison of the predicted average probability of tumor cell survival with the three models, the complete model, the spherical cap approximation model, and the model neglecting edge effect plotted for (A) different constant values of *r* with changing values of *K* and for (B) different constant values of *K* with changing values of *r/R*.
(TIF)

Figure S4 **Three-dimensional surface plot depicting probability of tumor cell survival.** Relationship between average probability of tumor cell survival and viral parameters *r/R* and *K*. Values of *r/R* and *K* have been converted to standard units of focus diameter (cell diameters) and percent of tumor cells infected at time zero of foci expansion respectively. The plot predicts viral parameter thresholds beyond which rapid decreases in survival probabilities occur with minimal changes in *r* or *K*.
(TIF)

Figure S5 **Model parameter** ***r*** **is dependent on tumor type and influences therapeutic efficacy.** Immunocompromised SCID mice bearing U266 myeloma tumors or immunocompetent mice bearing syngeneic 5TGM1 myeloma tumors were injected with a single IV dose of VSV (1×10^7^ and 1×10^8^ TCID_50_ respectively). Tumors were harvested at 24 or 48 hr post treatment as indicated and analyzed by immunofluorescence for VSV antigens (green in U266 tumor, red in 5TGM1 tumor) and tumor nuclei (blue). Immunofluorescence images show small, dispersed foci of infection with restricted expansion in U266 myeloma tumors, with larger, rapidly expanding and converging foci in 5TGM1 myeloma tumors at the same time point. Different tumor systems (5TGM1 compared to U266 myeloma tumors) with comparable foci density but vastly different foci diameters corresponded to regression of tumors with large foci and no regression in those with small foci, demonstrating the influence of foci diameter on therapeutic efficacy.
(TIF)

Table S1 **Components of the approximate spherical cap model derivation.**
(DOCX)

File S1 **Supplemental text describing mathematical derivation and approximations.**
(DOC)
