# Supplementary figures and images for: Mathematical Model for Radial Expansion and Conflation of Intratumoral Infectious Centers Predicts Curative Oncolytic Virotherapy Parameters
Source: PLoS One. 2013 Sep 11;8(9):e73759. doi: 10.1371/journal.pone.0073759 (PMC3770695; doi:10.1371/journal.pone.0073759)

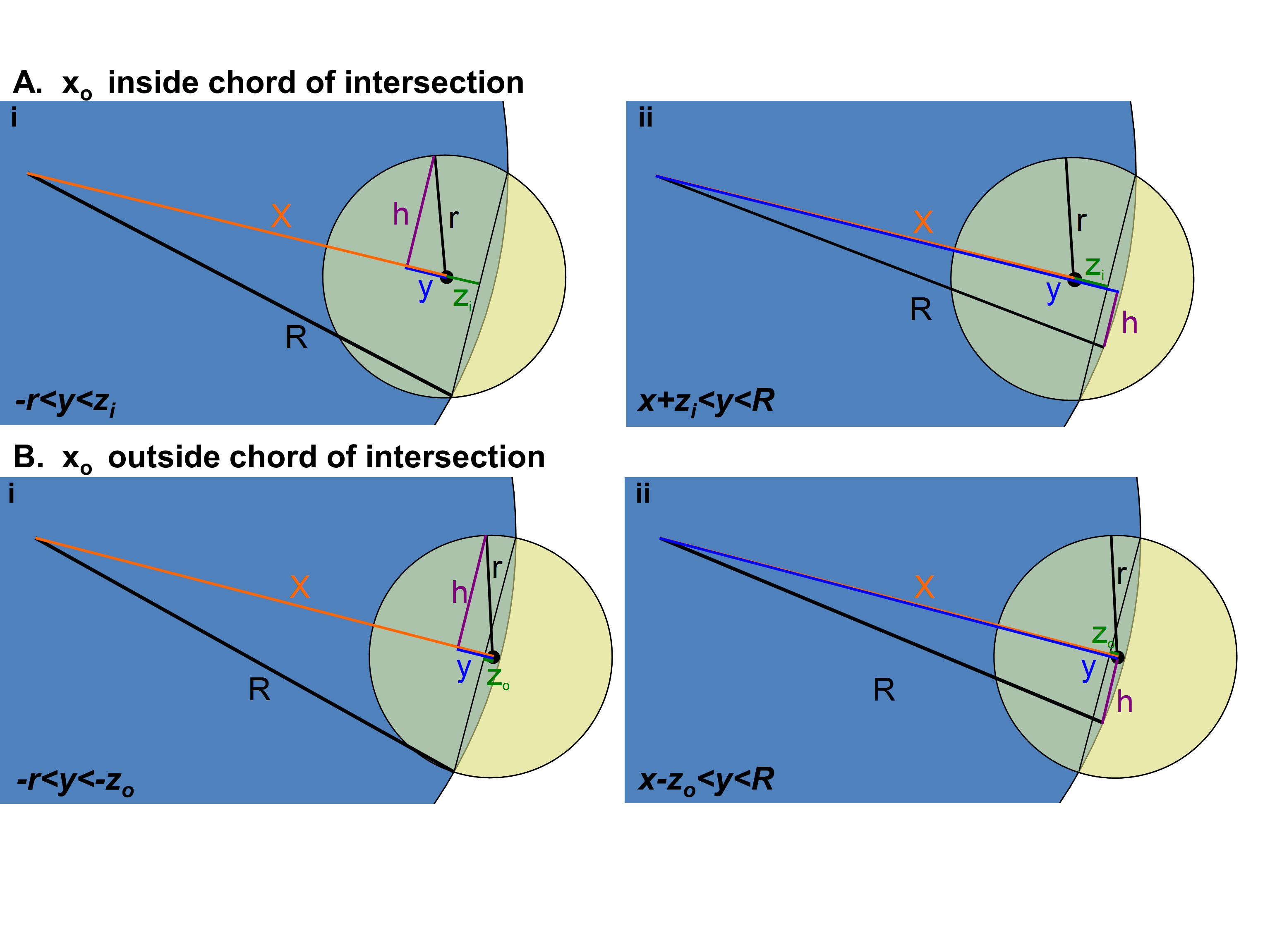

Supplement: Figure S1 — Modeling parameters for determination of lens of vulnerability volume. The volume of the lens created by the overlap of spheres of radii R and r is determined for two unique cases, (A) when xo lies inside the chord of intersection and (B) when xo lies outside the chord of intersection but within the tumor. Lens volume is determined by the sum of two spherical cap volumes using integration of circular disks of radius h and height dy for all values of y for each cap; (i) one cap generated by the curvature of sphere radius r and the chord of intersection, and (ii) one cap generated by the curvature of sphere radius R and the chord of intersection. (TIF) [file pone.0073759.s001.tif]

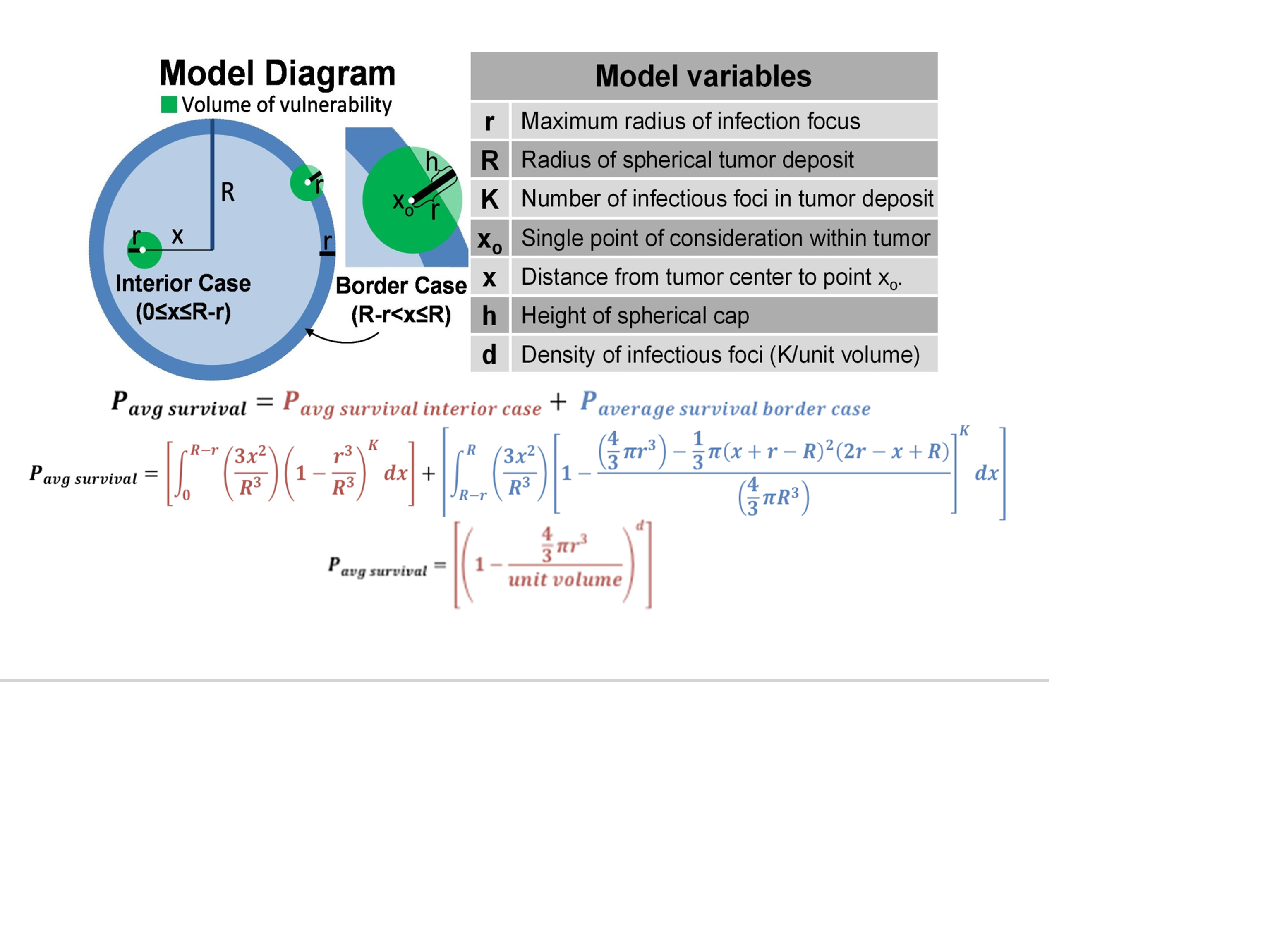

Supplement: Figure S2 — Mathematical model diagram of spherical cap approximation. Diagram of a spherical tumor depicting volumes of vulnerability for points within the interior and border case. Inset depicts the spherical cap approximation for calculating edge-effect. The model is presented as the average survival of any point xo in the spherical tumor radius R after expansion of K foci to maximum radius r. A simplified model neglecting edge-effect completely can be written in terms of foci per unit volume, d, and maximum radius r. (TIF) [file pone.0073759.s002.tif]

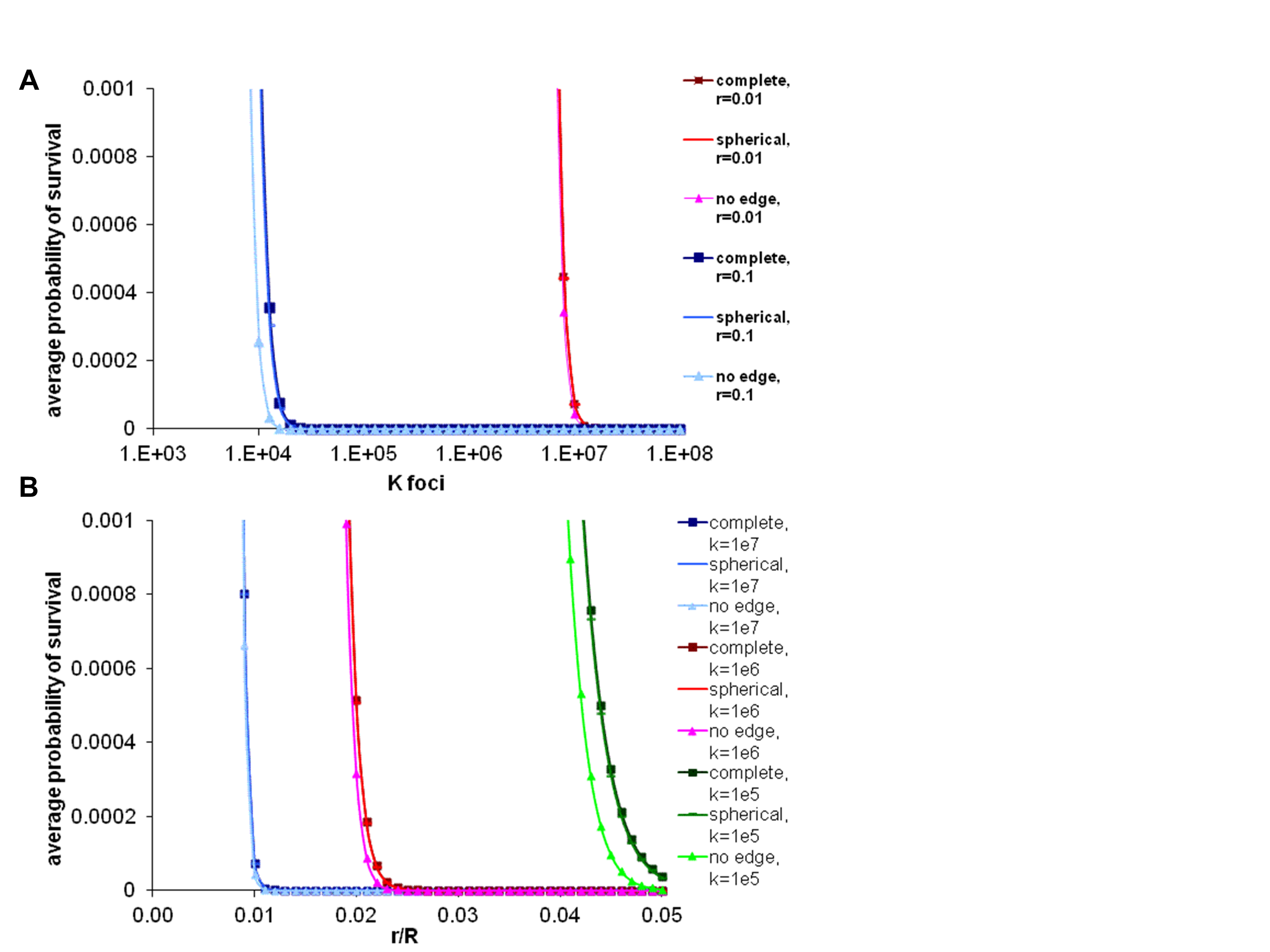

Supplement: Figure S3 — The influence of edge effect on model prediction of tumor survivability becomes increasingly negligible as viral parameters r/R and K increase. Comparison of the predicted average probability of tumor cell survival with the three models, the complete model, the spherical cap approximation model, and the model neglecting edge effect plotted for (A) different constant values of r with changing values of K and for (B) different constant values of K with changing values of r/R. (TIF) [file pone.0073759.s003.tif]

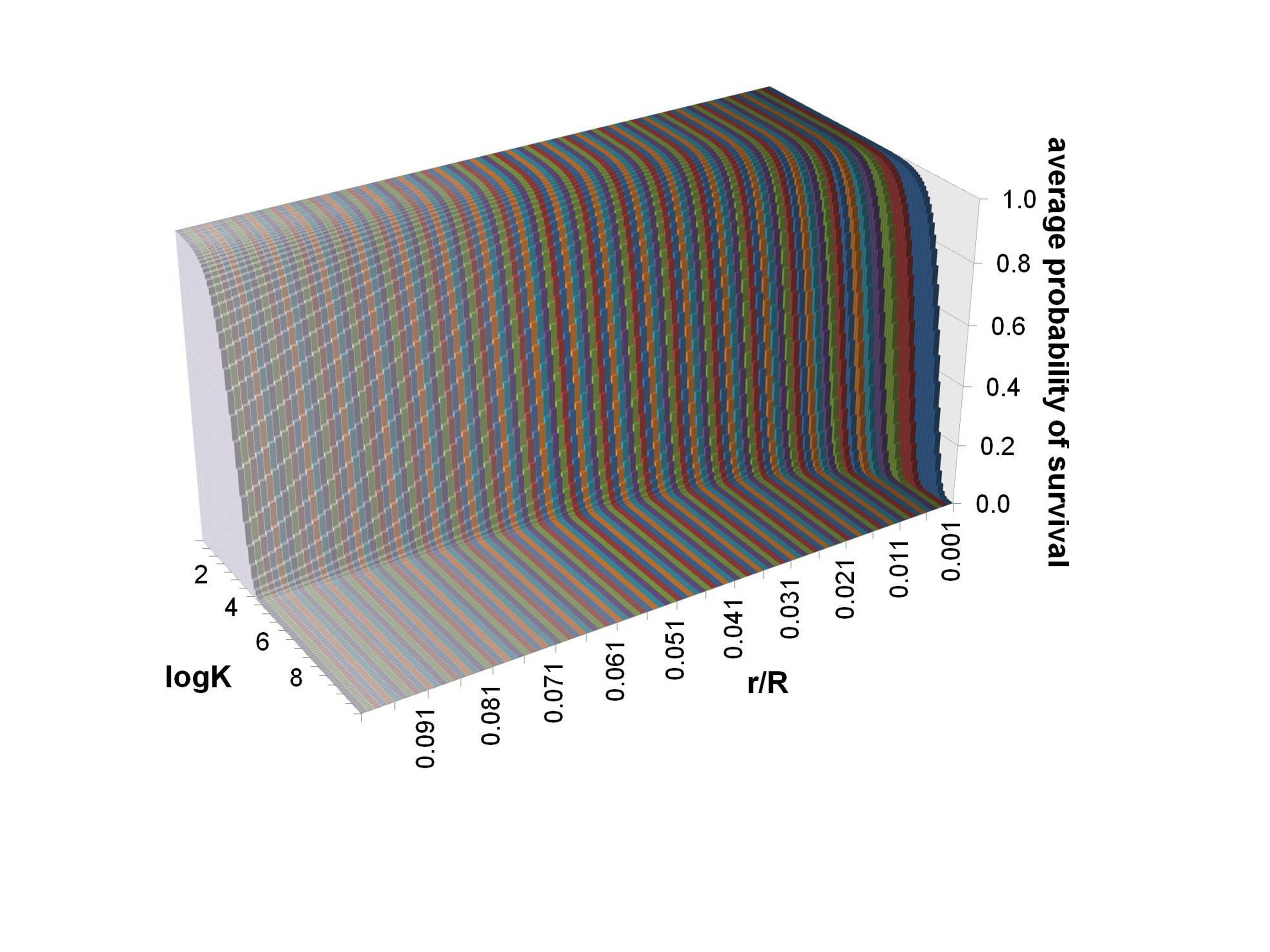

Supplement: Figure S4 — Three-dimensional surface plot depicting probability of tumor cell survival. Relationship between average probability of tumor cell survival and viral parameters r/R and K. Values of r/R and K have been converted to standard units of focus diameter (cell diameters) and percent of tumor cells infected at time zero of foci expansion respectively. The plot predicts viral parameter thresholds beyond which rapid decreases in survival probabilities occur with minimal changes in r or K. (TIF) [file pone.0073759.s004.tif]

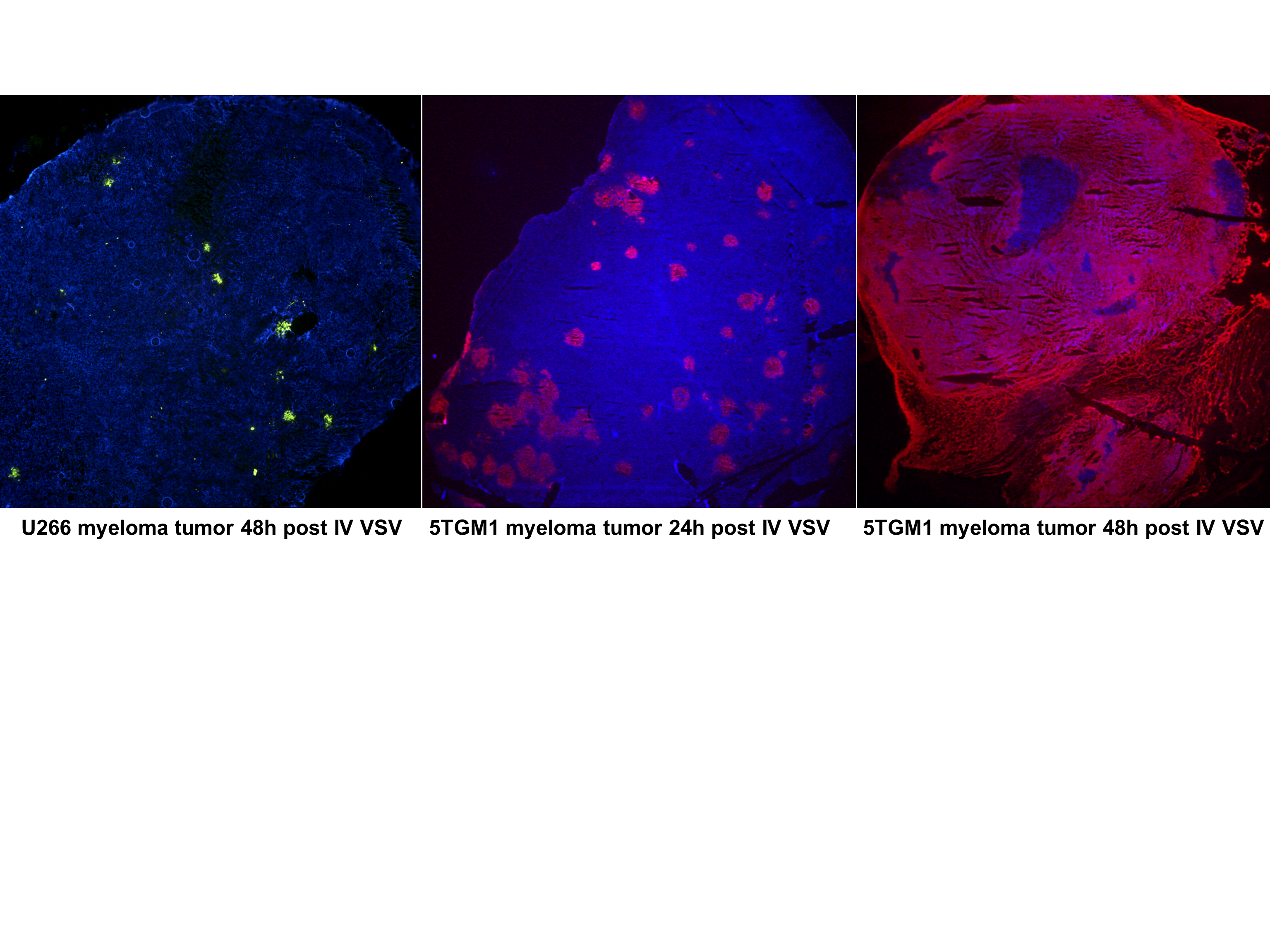

Supplement: Figure S5 — Model parameter r is dependent on tumor type and influences therapeutic efficacy. Immunocompromised SCID mice bearing U266 myeloma tumors or immunocompetent mice bearing syngeneic 5TGM1 myeloma tumors were injected with a single IV dose of VSV (1×10^7^ and 1×10^8^ TCID50 respectively). Tumors were harvested at 24 or 48 hr post treatment as indicated and analyzed by immunofluorescence for VSV antigens (green in U266 tumor, red in 5TGM1 tumor) and tumor nuclei (blue). Immunofluorescence images show small, dispersed foci of infection with restricted expansion in U266 myeloma tumors, with larger, rapidly expanding and converging foci in 5TGM1 myeloma tumors at the same time point. Different tumor systems (5TGM1 compared to U266 myeloma tumors) with comparable foci density but vastly different foci diameters corresponded to regression of tumors with large foci and no regression in those with small foci, demonstrating the influence of foci diameter on therapeutic efficacy. (TIF) [file pone.0073759.s005.tif]
