# Supplementary material for: Mathematical Model for Radial Expansion and Conflation of Intratumoral Infectious Centers Predicts Curative Oncolytic Virotherapy Parameters
Source: PLoS One. 2013 Sep 11;8(9):e73759. doi: 10.1371/journal.pone.0073759 (PMC3770695; doi:10.1371/journal.pone.0073759)
Supplement: Table S1 — Components of the approximate spherical cap model derivation. (DOCX) [file pone.0073759.s006.docx]

|  | **Interior case** | **Border Case** |
| --- | --- | --- |
| **Volume of vulnerability** | $\frac{4}{3}\pi r^{3}$ | $\left(\frac{4}{3}\pi r^{3}\right)-\left[\frac{1}{3}\pi\left(x+r-R\right)^{2}\left(2r-x+R\right)\right]$ |
| **Proportion of tumor vulnerable** | $\frac{\frac{4}{3}\pi r^{3}}{\frac{4}{3}\pi R^{3}}=\frac{r^{3}}{R^{3}}$ | $\frac{\left(\frac{4}{3}\pi r^{3}\right)-\left[\frac{1}{3}\pi\left(x+r-R\right)^{2}\left(2r-x+R\right)\right]}{\left(\frac{4}{3}\pi R^{3}\right)}$ |
| **Proportion not vulnerable** | $1-\left(\frac{r^{3}}{R^{3}}\right)$ | $1-\frac{\left(\frac{4}{3}\pi r^{3}\right)-\left[\frac{1}{3}\pi\left(x+r-R\right)^{2}\left(2r-x+R\right)\right]}{\left(\frac{4}{3}\pi R^{3}\right)}$ |
| **Probability of escaping *K* foci** | $\left[1-\left(\frac{r^{3}}{R^{3}}\right)\right]^{K}$ | $\left[1-\frac{\left(\frac{4}{3}\pi r^{3}\right)-\left[\frac{1}{3}\pi\left(x+r-R\right)^{2}\left(2r-x+R\right)\right]}{\left(\frac{4}{3}\pi R^{3}\right)}\right]^{K}$ |
| **Probability of consideration and escaping *K* foci** | $\int_{0}^{R-r} \left(\frac{3x^{2}}{R^{3}}\right)\left[1-\left(\frac{r^{3}}{R^{3}}\right)\right]^{K}dx$ | $\int_{R-r}^{R} \left(\frac{3x^{2}}{R^{3}}\right)\left[1-\frac{\left(\frac{4}{3}\pi r^{3}\right)-\left[\frac{1}{3}\pi\left(x+r-R\right)^{2}\left(2r-x+R\right)\right]}{\left(\frac{4}{3}\pi R^{3}\right)}\right]^{K}dx$ |
