# Supplementary material for: Mathematical Model for Radial Expansion and Conflation of Intratumoral Infectious Centers Predicts Curative Oncolytic Virotherapy Parameters
Source: PLoS One. 2013 Sep 11;8(9):e73759. doi: 10.1371/journal.pone.0073759 (PMC3770695; doi:10.1371/journal.pone.0073759)
Supplement: File S1 — Supplemental text describing mathematical derivation and approximations. (DOC) [file pone.0073759.s007.doc]

**File S1. Supplemental text describing mathematical derivation and approximations.**

**Supplemental Text**

**Mathematical Derivations**

The ***interior lens*** volume is determined by first defining the distance of *xo* from the chord of intersection, *zi*, in terms of the tumor radius, *R*, focus radius, *r*, and distance from the center of the tumor, *x*. Using Pythagorean Theorem, the height of the circular disk, *h*, can be defined as

$$\left(x+z_{i}\right)^{2} +\text{ }h^{2}=R^{2}$$

$$z_{i}^{2}+h^{2}=r^{2}$$

Combining these to solve for *zi* gives $z_{i}=\frac{(R^{2}-r^{2}-x^{2})}{2x}$

We then integrate the volume of circular disks of height *h*, with thickness of *dy* over the entire lens when *–r<y<zi* and for *zi<y<R* where *y* is the position along the same axis as distances *x* and *z* relative to the position of *xo*­.

$$\int_{-r}^{z_{i}} \pi(r^{2}-y^{2})dy+\int_{x+z_{i}}^{R} \pi(R^{2}-y^{2})dy$$

The similar formulation is done for the ***outer lens*** volume using the distance from the chord of intersection *zo*: Using Pythagorean Theorem, the height of the circular disk, *h*, can be defined as

$$\left(x-z_{o}\right)^{2} +\text{ }h^{2}=R^{2}$$

$$z_{o}^{2}+h^{2}=r^{2}$$

Combining these to solve for *zo* gives $z_{o}=\frac{(-R^{2}+r^{2}+x^{2})}{2x}$

We then integrate the volume of circular disks of height *h*, with thickness of *dy* over the entire lens when *–(r+zo)<y<-zo*and for –*zo<y<R* where *y* is the position along the same axis as distances *x* and *z* relative to the position of *xo*­.

$$\int_{-r}^{-z_{o}} \pi(r^{2}-y^{2})dy+\int_{x-z_{o}}^{R} \pi(R^{2}-y^{2})dy$$

*Model Approximation*

For the spherical cap model (rather than lens), the volume of a cap is first defined by its height, *h*, which is a function of *R* and *r*. The subsequent volume of vulnerability is the volume of the cap subtracted from the respective sphere of vulnerability (table S1).

$$\begin{matrix} V_{cap}=\frac{1}{3}\pi h^{2}\left(3r-h\right) \\ h=\left(x+r\right)-R \\ \end{matrix}$$
